# Evaluation of the efficacy of LAMP-based SARS-CoV-2 detection with simple RNA extraction from nasopharyngeal swabs: A prospective observational study

**DOI:** 10.1371/journal.pone.0260732

**Published:** 2021-12-14

**Authors:** Masaki Karino, Mizuki Harada, Chihiro Yamada, Kyoko Fukuoka, Megumi Sugo, Hiroyuki Hanada, Daisaku Masuda, Shingo Adachi, Shota Nakao, Masayuki Seki, Masaya Yamato, Shizuya Yamashita, Toru Takano

**Affiliations:** 1 Department of Clinical Laboratory, Rinku General Medical Center, Izumisano, Osaka, Japan; 2 Rinku Wellness Research Center, Rinku General Medical Center, Izumisano, Osaka, Japan; 3 Department of Cardiology, Rinku General Medical Center, Izumisano, Osaka, Japan; 4 Senshu Trauma and Critical Care Center, Rinku General Medical Center, Izumisano, Osaka, Japan; 5 Departments of General Internal Medicine and Infectious Diseases, Rinku General Medical Center, Izumisano, Osaka, Japan; 6 Departments of Diabetes/Endocrinology, Rinku General Medical Center, Izumisano, Osaka, Japan; University of Helsinki: Helsingin Yliopisto, FINLAND

## Abstract

The Loopamp SARS-CoV-2 Detection Kit is used for the detection of severe acute respiratory syndrome coronavirus 2 (SARS-CoV-2). Loop-mediated isothermal amplification (LAMP) is based on a measurement principle that can be used with a relatively simple device. Detection using this kit requires viral RNA extraction from samples with the QIAGEN QIAamp Viral Mini Kit (QIAGEN extraction) or the Loopamp Viral RNA Extraction Kit (Eiken extraction), which are recommended by the manufacturer. However, the efficacy of LAMP-based SARS-CoV-2 detection using these extraction methods has not been compared. In this study, we aimed to compare the results of genome extraction and detection from nasopharyngeal swab samples using the QIAGEN and Eiken extraction kits. The present study involved patients who presented to the Rinku General Medical Center with suspected COVID-19 (25 positive and 26 negative cases). A comparison of the results obtained using each extraction method with those obtained via PCR showed that the positive, negative, and overall concordance rates between QIAGEN extraction and PCR were 96.0% (24/25 samples), 100% (26/26), and 98.0% (50/51; κ = 0.96, 95% CI = 0.69–1.00), respectively. Results with Eiken extraction were also favorable, with positive, negative, and overall concordance rates of 88.0% (22/25), 100% (26/26), and 94.1% (48/51; κ = 0.88, 95% CI = 0.61–1.00), respectively. Favorable results were obtained using both QIAGEN and Eiken extraction kits. Since Eiken extraction can be completed in a few minutes, it enables prompt and reliable testing for SARS-CoV-2 detection.

## Introduction

In December 2019, a large number of pneumonia cases of unknown cause occurred mainly in Wuhan City, Hubei Province, People’s Republic of China, and a novel coronavirus, later named severe acute respiratory syndrome coronavirus 2 (SARS-CoV-2), was detected in these pneumonia cases in January, 2020 [1]. Acute respiratory syndrome caused by SARS-CoV-2, which is now a global pandemic, is referred to as coronavirus disease-2019 (COVID-19). We have been accepting patients with COVID-19 at our hospital since the early stages of the introduction of SARS-CoV-2 into Japan. Initially, reverse transcription-polymerase chain reaction (RT-PCR), performed as an administrative test in other facilities, was the only method for diagnosis confirmation; however, it took at least two days after sample submission to obtain test results. Subsequently, although RT-PCR tests became available at our hospital from March 19, 2020, it took more than 24 h to obtain the results, rendering it difficult to report rapidly, especially when a large number of tests had to be performed simultaneously. On April 10, 2020, the Loopamp COVID-19 (SARS-CoV-2) Detection Kit was launched by Eiken Chemical Co. Ltd. (Tokyo, Japan). This kit uses the loop-mediated isothermal amplification (LAMP) method, a gene amplification reaction that progresses in an isothermal state, using four primers that recognize six regions in the nucleotide sequence of the target gene [2, 3]. For this procedure, pretreatment for viral RNA extraction using either of the following two kits is recommended: QIAamp Viral Mini Kit (QIAGEN, Hilden, Germany) and Loopamp Viral RNA Extraction Kit (Eiken Chemical). The former is a standard method for gene extraction, and it can efficiently concentrate the samples; however, it is time-consuming, and the procedures are complicated. In contrast, the latter involves operations that can be performed very quickly and easily; however, it yields a smaller volume of samples for use in the detection kit than the QIAGEN kit, which may produce false-negative results for samples with a low viral content. These differences suggest that the sensitivity of the LAMP analysis and the number of samples that can be tested in a day may differ depending on the pretreatment method. Although an initial assessment at our hospital suggested a high concordance between RT-PCR and LAMP analysis using the Loopamp Viral RNA Extraction Kit, how RNA extraction methods affect detection capability has not yet been reported. Moreover, the correlation between the results of RT-LAMP and days after onset of symptoms is not clear. Thus, in this study, we evaluated the performance of LAMP-based SARS-CoV-2 detection using the Loopamp Viral RNA Extraction Kit and clinical nasopharyngeal swab samples.

## Materials and methods

### Subjects

From each patient with COVID-19 diagnosed at Rinku General Medical Center (including suspected cases) between May 19, 2020 and January 31, 2021, who consented to the secondary use of samples (25 positive and 26 negative cases), two nasopharyngeal swabs (sponge-type swab TYPE S, Nipro, Tokyo, Japan) were collected and used as clinical samples. The protocol of this study was reviewed by the ethics committee of Rinku General Medical Center and approved by the Director of the hospital (No. 2020–003, entitled "Investigation of clinical usefulness of a COVID-19 detection kit"). The turnaround time (TAT), defined as the time from sample submission to completion of reporting of test results, was collected for all 430 samples (including saliva samples) submitted during the period from the introduction of the LAMP method to August 31, 2020.

### Detection of SARS-CoV-2

Of the two collected swabs, one was subjected to RNA extraction using the QIAGEN QIAamp Viral Mini Kit (hereinafter “QIAGEN extract”), and the other was subjected to RNA extraction using the Loopamp Viral RNA Extraction Kit (hereinafter “Eiken extract”). These two extracts were subjected to the following four assays:

#### 1) LAMP assay of the QIAGEN extract (hereinafter “Q-LAMP”)

QIAGEN extraction was performed by partially modifying the method described in the instruction manual for the kit. The sample (130 μL; a swab suspended in 1 mL of saline) was mixed with 560 μL of the buffer AVL/carrier RNA mixture and allowed to stand for 10 min. Subsequently, 10 μL of LightMix EAV RNA Extraction Control (TIB MOLBIOL, Berlin, Germany) was added as a positive control (target RNA). The final volume of the eluate was 60 μL (AVE buffer). The reaction and interpretation of the results of the LAMP assay were performed using the Loopamp Realtime Turbidimeter (Eiken Chemical), and measurements were performed according to the instructions in the package insert of the kit. Primer Mix 2019-nCoV (15 μL) and the sample or control solution (10 μL) were dispensed into the reaction tube, reacted with the dried RNA amplification reagent for 2 min, and mixed by inversion, and the measurement was initiated. For samples that showed a positive result, the threshold time (Tt) corresponding to the time of viral detection was recorded.

#### 2) LAMP assay of the Eiken extract (hereinafter “E-LAMP”)

According to the instruction manual, the swab sample was stirred up and down 10 times in the Loopamp Viral RNA Extraction Reagent and used as the extract. The LAMP assay was performed in the same manner as described above.

#### 3) RT-PCR assay of the QIAGEN extract (hereinafter “PCR”)

The QIAGEN extract prepared in Assay 1 was used as the sample and subjected to RT-PCR using the Cobas z 480 analyzer (Roche, Basel, Switzerland), LightMix Modular SARS-CoV (COVID-19) E-gene kit (TIB MOLBIOL), LightMix EAV RNA Extraction Control, and LightCycler Multiplex RNA Virus Master (Roche). A 20 μL reaction solution was prepared by mixing 4.9 μL of PCR grade water, 4 μL of RT-qPCR Reaction Mix, 0.5 μL each of the reagent mixtures (E-gene and EAV RNA), 0.1 μL of RT Enzyme Solution, and 10 μL of the QIAGEN extract and subjected to RT-PCR under the following reaction conditions: 55°C for 5 min, 95°C for 5 min, followed by 45 cycles of 95°C for 5 s, 60°C for 15 s, and 72°C for 15 s [4]. For positive samples, the threshold cycle (Ct) was calculated using the second derivative maximum method. EAV RNA was detected in all QIAGEN extracts, confirming the validity of the extraction procedure.

#### 4) LAMP assay of RNA-concentrated samples prepared from the Eiken extracts

The residue of the Eiken extract from Assay 2 was subjected to RNA concentration and purification using the QIAamp Viral RNA Mini Kit or the MagLEAD system (Precision System Science, Chiba, Japan), followed by the LAMP reaction. Using the QIAamp Viral RNA Mini kit, 140 μL of the Eiken extract was processed following the method described in Assay 1 (hereinafter “E-Q-LAMP”). Using the MagLEAD fully automated extraction system, 200 μL of the Eiken extract was loaded into the MagLEAD 6gC and MagDEA Dx SV 200 6gC systems and eluted in 50 μL. The extracts obtained via each method were processed to prepare LAMP reaction mixtures, as described in Assay 1 and the reaction was performed in triplicate (hereinafter “E-M-LAMP”).

### Data analysis

On the basis of the assay results obtained as described above, the concordance rate between Q-LAMP and E-LAMP was calculated using the PCR method as a control. For the samples with discrepant results between the PCR and LAMP-based methods, remeasurements were performed using the Q-LAMP (n = 1) and E-LAMP (n = 3) methods with the remaining samples.

Scatter plots were prepared for the Ct values determined using the PCR method, the Tt values for samples that tested positive via the Q-LAMP and E-LAMP methods, and the difference in Tt values determined using the two LAMP-based methods against the number of days elapsed from symptom onset to sample collection; the data were analyzed for any trend.

To examine the correlation between LAMP-Tt and PCR-Ct values, scatter plots were prepared for the Tt values determined using the E-LAMP method, Tt values determined using the Q-LAMP method, and the difference in Tt values determined using the two LAMP-based methods against the PCR-Ct values; the data were analyzed for any trend.

For statistical analysis, Cohen’s κ coefficient and 95% confidence interval (CI) were determined to evaluate the concordance between the detection methods. StatFlex version 7 (Artech, Osaka, Japan) was used for the statistical analysis.

## Results

### Comparison of viral detection between Q-LAMP and E-LAMP methods using RT-PCR as the control

The results for the Q-LAMP and PCR methods are presented in Table 1. The positive concordance rate was 96.0% (24/25 samples), negative concordance rate was 100% (26/26), and overall concordance rate was 98.0% (50/51) (κ = 0.96, 95% CI = 0.69–1.00). The sample with discrepant results had a PCR Ct value of 35.67. The results for the E-LAMP and PCR methods are presented in Table 1. The positive concordance rate was 88.0% (22/25 samples), the negative concordance rate was 100% (26/26), and the overall concordance rate was 94.1% (48/51) (κ = 0.88, 95% CI = 0.61–1.00). One of the samples with discrepant results between the PCR and E-LAMP methods also showed discrepant results between the PCR and Q-LAMP methods. The other two samples with discrepant results had PCR-Ct values of 27.26 and 31.26, respectively (Table 2). Compared with the Q-LAMP method, the E-LAMP method showed positive, negative, and overall concordance rates of 91.7%, 100%, and 96.1%, respectively (κ = 0.92, 95% CI = 0.65–1.00) (Tables 1 and 2). In any comparison among three assays, the kappa values were higher than 0.81 and there was "almost perfect" statistical agreement [5].

**Table 1:** Comparison of the results between LAMP and RT-PCR for the detection of SARS-CoV-2.

		Q-LAMP		Total	E-LAMP		Total
		Positive	Negative		Positive	Negative	
PCR	Positive	24	1	25	22	3	25
	Negative	0	26	26	0	26	26
Total		24	27	51	22	29	51

**Table 2:** Results of retests performed on three cases of dissociation between PCR and LAMP.

Sample No.	Days after onset of symptoms	PCR Ct value	Q-LAMP		E-LAMP			
			Test	Retest	Test	Retest		
R-001	21	27.26	34.6	−	−	−	−	−
R-038	9	31.26	17.5	17.4	−	−	23.2	31.6
R-044	12	35.67	−	25.8	−	−	−	−

In the cases where the LAMP assay showed positive results, the Tt value is presented.

### Analysis of three samples with discrepant results between the PCR and LAMP-based methods

The results of remeasurement (Q-LAMP and E-LAMP methods) and additional measurements (E-Q-LAMP and E-M-LAMP methods) of the three samples with discrepant results between the PCR and LAMP-based methods are presented in Tables 2 and 3, respectively. For sample R-001, all measurements and additional measurements were negative. For sample R-038, the measurement results were reproducible via the Q-LAMP method, and remeasurements using the E-LAMP method tested positive in two of the three replicates. Moreover, all triplicate measurements were positive using the E-Q-LAMP and E-M-LAMP methods. Sample R-044 tested positive via the Q-LAMP method, whereas all measurements using the E-LAMP and E-Q-LAMP methods yielded negative results. With the E-M-LAMP method, one of the triplicate measurements was positive.

**Table 3:** Results of additional tests with condensation of virus RNA for deviated cases.

Sample No.	E-Q-LAMP			E-M-LAMP		
R-001	−	−	−	−	−	−
R-038	19.3	18.2	19.1	24.7	23.1	22.1
R-044	−	−	−	−	29.2	−

In the cases where the LAMP assay showed positive results, the Tt value is presented.

### Relation between test-positivity and the number of days after onset of symptoms

**Fig 1:**
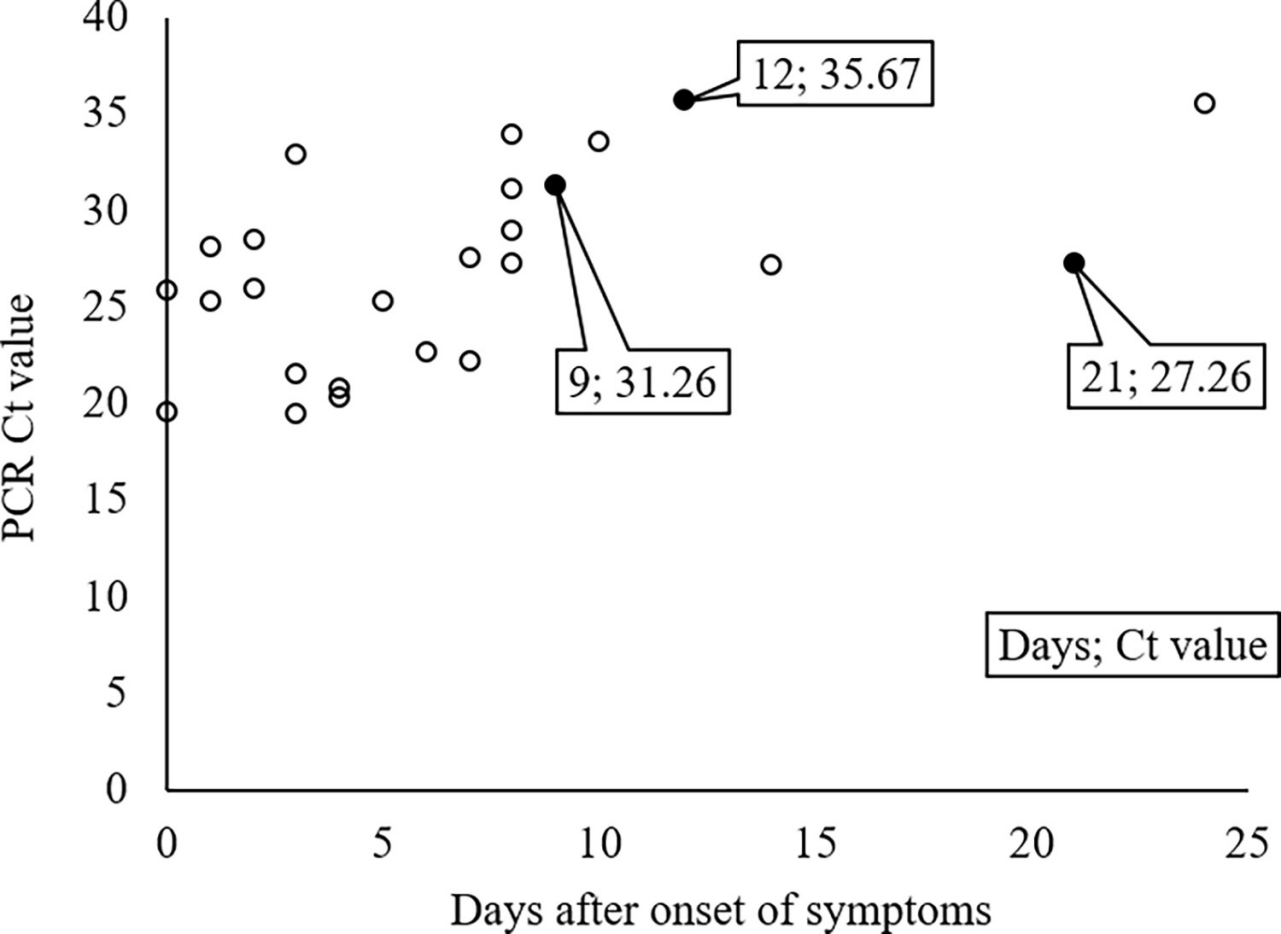
Relation between PCR and LAMP deviations and timing of specimen collection. For all PCR-positive cases, the Ct values of PCR and the days after the onset of symptoms were plotted. White and black circles indicate the E-LAMP-positive and -negative cases, respectively. The days after onset of symptoms and Ct values according to PCR for the E-LAMP-negative cases are indicated in the figure.

**Fig 2:**
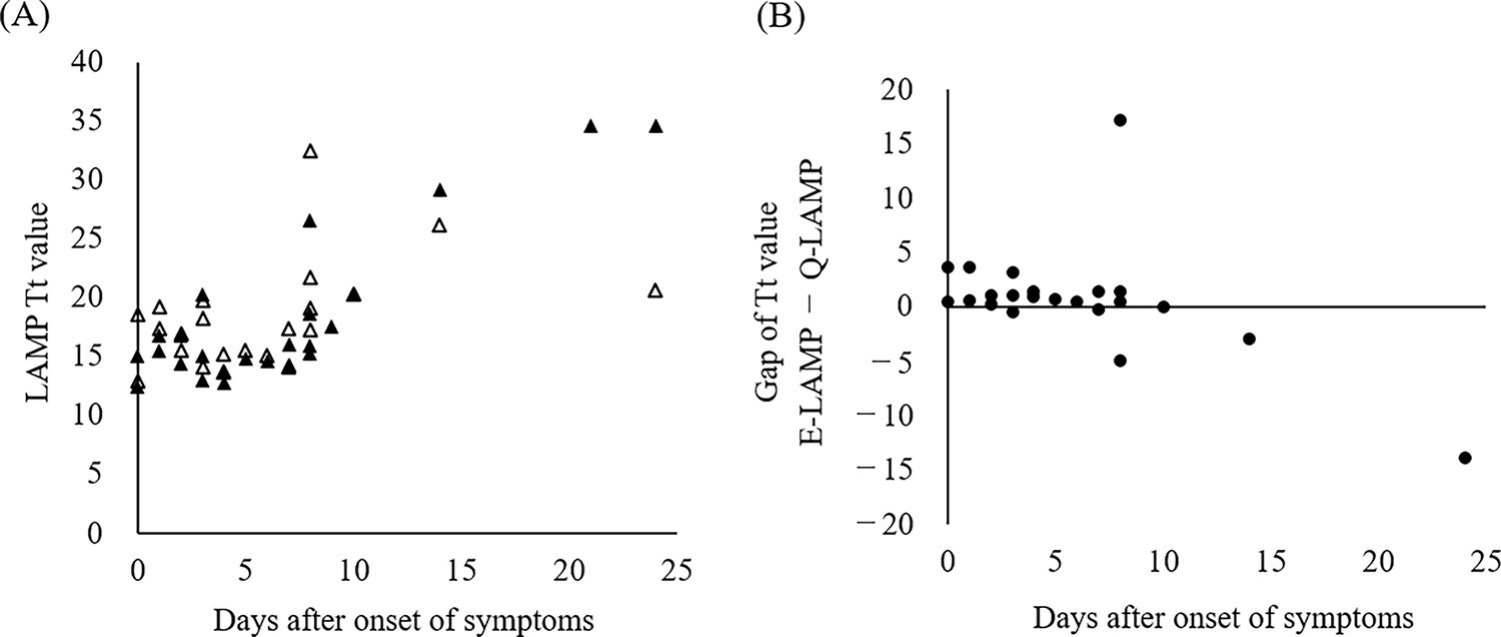
Trend for the Tt value with LAMP, with focus on the time since onset. (A) Tt values of LAMP and the number of days elapsed since the onset of disease in Q-LAMP-and E-LAMP-positive cases are shown. Black and white triangles indicate the Q-LAMP- and E-LAMP-positive cases, respectively. (B) The difference is calculated between the Tt values for LAMP performed using the two extraction methods and represented along with the days after onset of symptoms. Samples R-001, R-038, and R-044 were excluded (ref. Table 2).

For SARS-CoV-2-positive samples, scatter plots of the number of days from symptom onset to sample collection against the PCR-Ct value were prepared and presented, along with the results of the E-LAMP assay (Fig 1). All samples collected within 8 days after symptom onset tested positive using the E-LAMP method (19/19), while half of the samples collected 9 days after symptom onset tested negative (3/6). The sample that tested negative using the Q-LAMP method (R-044) was collected 12 days after symptom onset (Table 2). The relationship between the Tt values determined using the LAMP-based methods (LAMP-Tt) and the number of days after symptom onset is shown in Fig 2A. Most of the samples collected within 7 days after symptom onset exhibited LAMP-Tt values ≤20 min. To compare the Tt values between the E-LAMP and Q-LAMP methods, we calculated the difference in Tt values obtained using each method and plotted them as scatter plots against the number of days after symptom onset (Fig 2B). Samples collected within 7 days after symptom onset showed stable and similar Tt values between the two methods, while samples collected at later time points after symptom onset yielded more discrepant or false-negative results.

### Relationship between LAMP-Tt and PCR-Ct values

**Fig 3:**
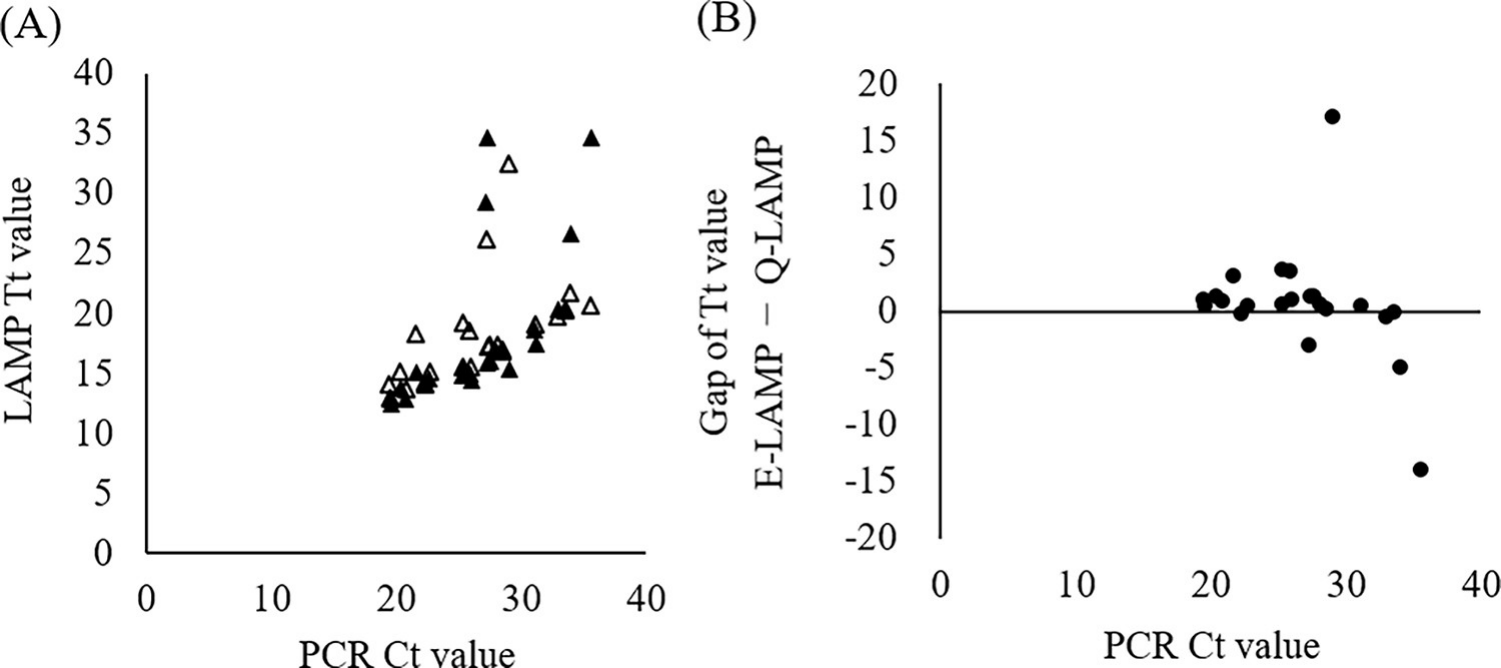
Correlation between the Ct value with PCR and Tt value with LAMP. (A) The Tt values with LAMP and Ct values with RT-PCR in Q-LAMP- and E-LAMP-positive cases are presented. Black and white triangles indicate the Q-LAMP- and E-LAMP-positive cases, respectively. (B) The gap in the Tt values calculated in 2B is shown along with the Ct value with PCR. Samples R-001, R-038, and R-044 were excluded (ref. Table 2).

Scatter plots of LAMP-Tt and PCR-Ct values are presented in Fig 3A. All samples with PCR-Ct values <27 exhibited Tt values ≤20 min, and the same trend was observed for samples with PCR-Ct values of approximately 30. To compare the Tt values between the E-LAMP and Q-LAMP methods, we calculated the difference in Tt values obtained using each method and plotted the values as scatter plots against the PCR-Ct values (Fig 3B). Samples with Ct values <30, which indicate a substantial viral load, showed similar values between the two methods; however, viral load quantification using RT-PCR was not performed in this study.

### TAT for LAMP-based methods

**Fig 4:**
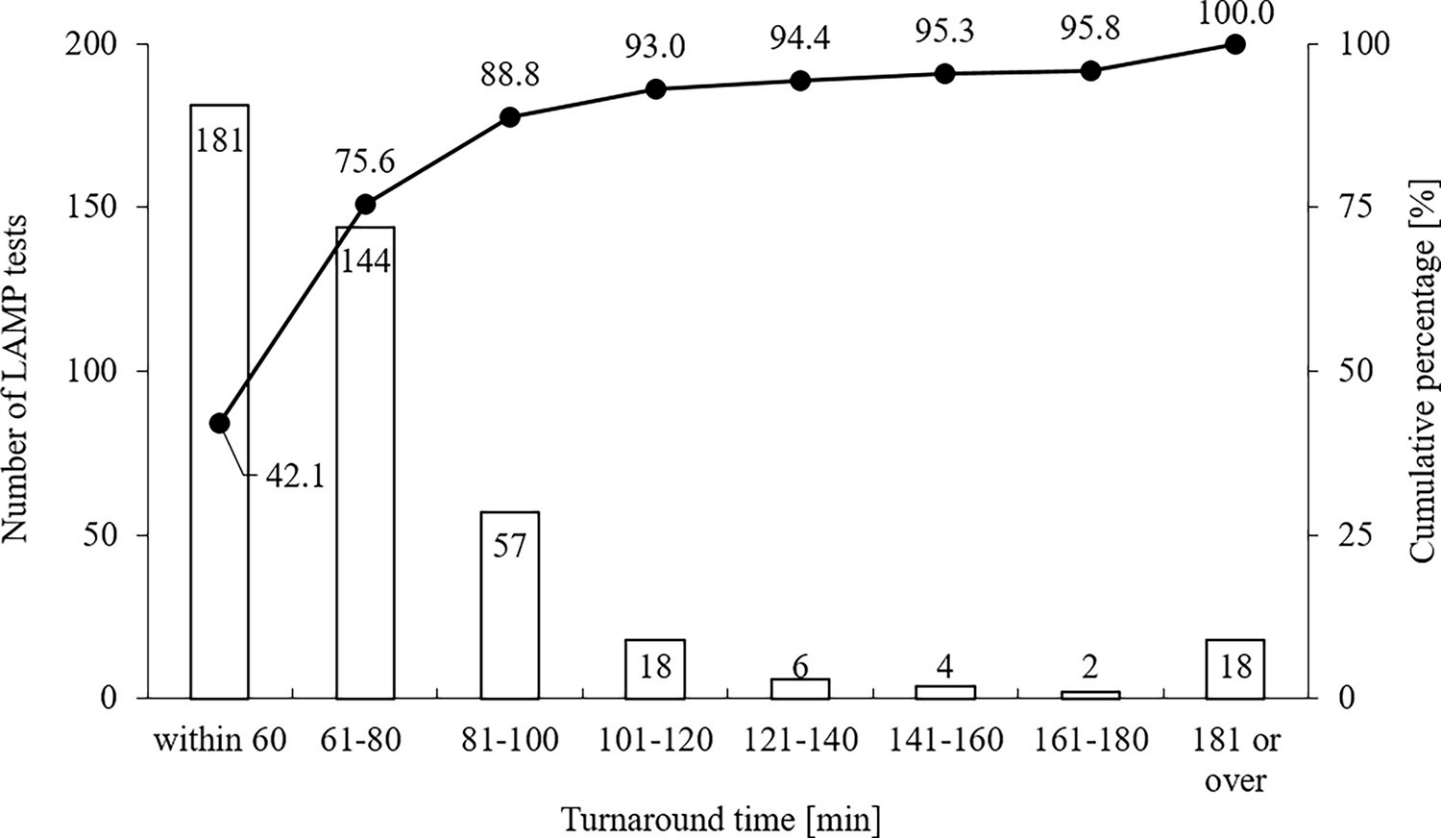
The turnaround time of the detection test for SARS-CoV-2 using E-LAMP. The number of LAMP tests per indicated TAT (bar graph; left axis) and the cumulative percentage of all samples (line graph; right axis) are presented.

The TAT data for samples submitted during the investigation period are presented in Fig 4, along with the cumulative percentage of samples for which results were reported. Of the 430 samples, 75.8% exhibited a TAT ≤80 min and 93.0% exhibited a TAT ≤120 min.

## Discussion

The results of this study demonstrate that the Q-LAMP method is highly concordant with the PCR method (Table 1). These results are similar to those reported previously [3], implying that the results were reproducible in our hospital. The sample with discrepant results between the Q-LAMP and PCR methods (R-044) tested positive upon remeasurement, exhibiting a concordance rate of 100%. This study compared different viral detection methods using the same extract, and the results showed that the performance of the Loopamp COVID-19 (SARS-CoV-2) Detection Kit is equivalent to that of the PCR-based detection kit. Similarly, the E-LAMP method using the simple Eiken extraction method showed good concordance (Table 1). However, an analysis of data from the samples with discrepant results between the E-LAMP and Q-LAMP methods suggested that caution should be exercised when making judgments based solely on the results of the E-LAMP assay (Table 2). In sample R-001, it took nearly 35 min before a positive reaction was detected using the Q-LAMP method. The result was not reproducible, and the reason for this was unknown. All measurements using the E-LAMP method and additional measurements of concentrated extracts yielded negative results, which was likely because of the difference between swabs used in each measurement (Tables 2 and 3). Sample R-038 was subjected to remeasurement using the E-LAMP method in triplicate and yielded a positive result (in two of three replicates). With the E-LAMP method, the sample tested positive only in two of the four replicates and thus was assumed to contain a low viral load. In fact, the additional measurement for the Eiken extract concentrated via QIAGEN extraction and MagLEAD extraction showed positive results in all three replicates (Tables 2 and 3). Additionally, with the E-M-LAMP method, sample R-044 tested positive in one of three replicates; with the E-LAMP method, the Eiken extract tested negative in all replicates. These results indicated condensation of virus RNA (Tables 2 and 3) and suggest that for samples that were below the detection sensitivity via the E-LAMP method, the concentration of the Eiken extracts is useful for confirming the results of the E-LAMP method.

Although several other LAMP-based reagents for detection of SARS-CoV-2 were reported in the early stages of the pandemic, total viral RNA required complicated extraction process [6, 7]. Subsequently, a LAMP-based assay was developed that allowed testing using a simplified protocol with pre-heating without extraction [8–10]. Evaluation of RT-LAMP in which pre-heating was adopted instead of extraction showed a decline in detection performance for samples when Ct values for reference RT-PCR (rPCR) were 30 or greater [9, 10]. Our results indicated similar correlation as in a previous report. For samples with rPCR Ct values below 30, E-LAMP positivity was 17/18 (94.4%), and for samples with rPCR Ct values above 30, it was 5/7 (71.4%) (Fig 3 and S1 Table).

When the assay results were analyzed with a focus on the day of symptom onset, the samples with discrepant results between the PCR- and LAMP-based methods tended to be collected at later times after symptom onset (i.e., 9, 12, and 21 days; Fig 1). In contrast, for samples collected at earlier time points after symptom onset, which were assumed to have a relatively higher viral load, the E-LAMP and Q-LAMP methods demonstrated an equivalent detection performance (Figs 2 and 3), suggesting the effectiveness of the E-LAMP method for testing at this stage owing to its simplicity and speed of operation. For samples collected at later or unknown time points after symptom onset, it is desirable to extract RNA from the Eiken extract, concentrate it, and perform a confirmatory test using a LAMP- or PCR-based method. In many infected patients, the viral load of SARS-CoV-2 becomes detectable 2–3 days before symptom onset, reaches a peak around the day of symptom onset, and decreases in approximately 7–8 days, and the duration of SARS-CoV-2 infectivity is reported to be 10–15 days depending on the severity of the disease and immune status [11]. Moreover, despite the involvement of personnel from non-specialty departments, the test results were reported within 80 min for 75.6% of the samples, enabling a rapid response to testing (Fig 4). Given these factors, this study shows that E-LAMP assay is one of superior point-of-care tests (POCTs) for screening of infectious patients.

Currently, valuable POCTs using RT-PCR and nicking enzyme amplification reaction (NEAR) as measurement principles are utilized for the diagnosis of COVID-19 [12–14]. In particular, the Cepheid Xpert Xpress assay was reported to be superior than other assays for both assay limit-of-detection and clinical performance [14]. However, its characteristics, such as high costs, maximum throughput, and instrument size, may pose a problem. The LAMP-based assay has advantages, such as low cost, visual determination without special equipment (not used in this study), and increase in maximum throughput by adding more modules (up to 96 cases for the Loopamp Realtime Turbidimeter) [6–8, 10, 15]. It is necessary to understand the characteristics of each POCT and select the appropriate one.

There are several limitations to this study. First, because only nasopharyngeal samples were included in this study, the detection performance for saliva specimen using Eiken extraction remains unclear. Self-collected saliva is less likely to result in the exposure of medical staff to infectious virus, and is, therefore, a preferred sample that has previously been studied [16, 17]. The test specimen should be selected with sufficient consideration of its compatibility with POCT. Second, although in RT-PCR, used as a reference, the viral load can be estimated in terms of the Ct value, it does not quantify the load; therefore, we could not quantitatively evaluate the limit-of-detection. However, as mentioned above, we were able to evaluate the method as a screening test for infected people who may have secondary infection. Finally, because TAT analysis was based on the results of a practical clinical laboratory test, excessively prolonged TATs were obtained in anomalous cases because of nonspecific reactions or other reasons, which could not be interpreted (Fig 4).

The present study shows that the LAMP assay with Eiken extraction enables highly sensitive detection of SARS-CoV-2 from nasopharyngeal swabs collected within 7–8 days of the disease. Using this method, the status of SARS-CoV-2 infection can be determined rapidly and reliably.

## Supporting information

S1 Table Data set that this work analysed. (XLSX)
